# Deep Learning in Neovascular Age-Related Macular Degeneration

**DOI:** 10.3390/medicina60060990

**Published:** 2024-06-17

**Authors:** Enrico Borrelli, Sonia Serafino, Federico Ricardi, Andrea Coletto, Giovanni Neri, Chiara Olivieri, Lorena Ulla, Claudio Foti, Paola Marolo, Mario Damiano Toro, Francesco Bandello, Michele Reibaldi

**Affiliations:** 1 Division of Ophthalmology, Department of Surgical Sciences, University of Turin, Via Verdi, 8, 10124 Turin, Italy; sonia.serafino@unito.it (S.S.); federico.ricardi@unito.it (F.R.); andrea.coletto@unito.it (A.C.); giovanni.neri@unito.it (G.N.); chiara.olivieri@unito.it (C.O.); lorena.ulla@unito.it (L.U.); claudio.foti1@gmail.com (C.F.); michele.reibaldi@unito.it (M.R.); 2 Department of Ophthalmology, “City of Health and Science” Hospital, 10126 Turin, Italy; 3 Eye Clinic, Public Health Department, University of Naples Federico II, 80138 Naples, Italy; mariodamiano.toro@unina.it; 4 Department of Ophthalmology, Vita-Salute San Raffaele University, 20132 Milan, Italy; bandello.francesco@hsr.it; 5 IRCCS San Raffaele Scientific Institute, 20132 Milan, Italy

**Keywords:** age-related macular degeneration, optical coherence tomography, neovascularization, neovascular age-related macular degeneration, artificial intelligence, deep learning, biomarker

## Abstract

*Background and objectives*: Age-related macular degeneration (AMD) is a complex and multifactorial condition that can lead to permanent vision loss once it progresses to the neovascular exudative stage. This review aims to summarize the use of deep learning in neovascular AMD. *Materials and Methods*: Pubmed search. *Results*: Deep learning has demonstrated effectiveness in analyzing structural OCT images in patients with neovascular AMD. This review outlines the role of deep learning in identifying and measuring biomarkers linked to an elevated risk of transitioning to the neovascular form of AMD. Additionally, deep learning techniques can quantify critical OCT features associated with neovascular AMD, which have prognostic implications for these patients. Incorporating deep learning into the assessment of neovascular AMD eyes holds promise for enhancing clinical management strategies for affected individuals. *Conclusion*: Several studies have demonstrated effectiveness of deep learning in assessing neovascular AMD patients and this has a promising role in the assessment of these patients.

## 1. Introduction

Age-related macular degeneration (AMD) represents a major cause of visual impairment worldwide, with its prevalence steadily rising owing to demographic shifts and the continuous growth of the elderly population. Projections indicate that by 2040, approximately 288 million individuals will be affected [1]. Numerous factors are involved in the pathogenesis of this disease, including aging, genetic predisposition, and environmental factors such as smoking, diet, higher body mass index (BMI), and cardiovascular disease. Consequently, AMD presents itself as a complex array of disorders with a multifactorial etiology [2]. Regardless of the etiology, AMD is ultimately considered as a disease characterized by the dysfunction of the unit comprised of photoreceptors, retinal pigment epithelium (RPE), Bruch’s membrane and choriocapillaris [3,4,5,6,7,8,9,10,11,12,13].

A multimodal imaging approach is essential in diagnosing and treating AMD, utilizing several modalities including fluorescein angiography (FA), indocyanine green angiography (ICGA), structural optical coherence tomography (OCT), and OCT angiography (OCTA). With approximately 30 million scans per year, structural OCT provides high-resolution images of the retinal choroidal structures and anatomy, making it the most important imaging employed in ophthalmology for follow-up and predicting treatment response [14].

The advanced form of AMD is characterized by the development of either macular neovascularization (MNV) or geographic atrophy (GA), which are both responsible for the majority of cases of visual impairment in eyes with AMD [15,16,17,18,19,20,21,22]. MNV is characterized by the proliferation of abnormal blood vessels and subsequent leakage due to their immature vascular network. The neovascularization can manifest in various retinal layers: between the RPE and Bruch’s membrane (i.e., Type 1 MNV, the predominant subtype), within the sub-retinal space (i.e., Type 2 MNV), and within the intra-retinal space (i.e., Type 3 MNV or retinal angiomatous proliferation—RAP, the second most common subtype) [23,24]. Type 1 and 2 MNV originate from the choroid, whereas Type 3 MNV arises from the deep retinal capillary plexus or the deep vascular complex, extending downward toward the RPE [21,25,26,27,28,29,30].

In this review, we will delve into the primary biomarkers linked to neovascular AMD. The discovery of these biomarkers has been facilitated by advancements in imaging technologies, notably the advent of structural OCT and OCTA. Furthermore, we will explore the utilization of deep learning algorithms to detect and measure these biomarkers. This aspect holds significant clinical implications, as deep learning algorithms streamline the process of identifying and quantifying these biomarkers, addressing the time constraints associated with manual assessment and reducing reliance on the expertise of individual readers.

**Figure 1:**
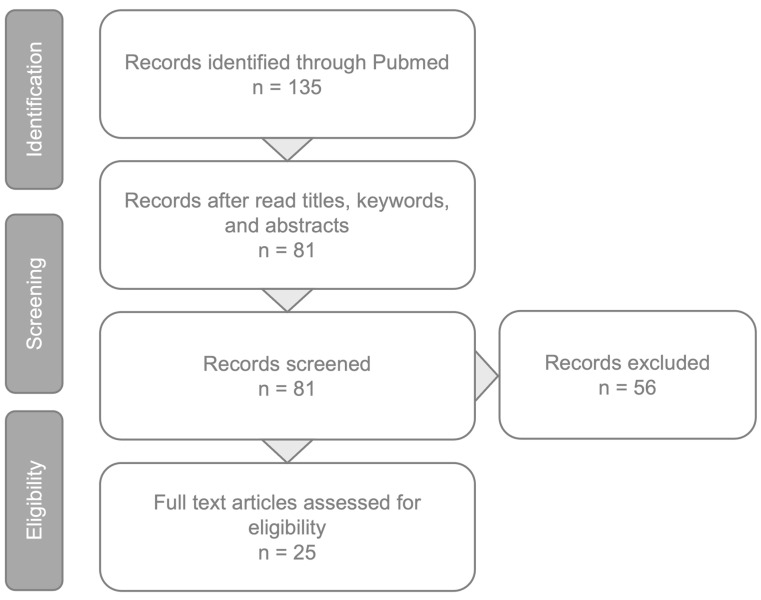
Article review and selection process.

A PubMed engine search was conducted utilizing the keyword “neovascular AMD” in conjunction with “artificial intelligence” and “deep learning”. All studies published in between January 2016 and February 2024 were reviewed. The relevant publications identified through this process and selected by the authors have been incorporated into this review (Figure 1).

## 2. OCT Biomarkers in Neovascular AMD

Biomarkers are considered as morphological signs which are useful in clinical practice in the diagnostic process, assessing the disease progression and predicting or monitoring the effect of therapeutic decisions. In this scenario, structural OCT is greatly useful as it can identify retinal biomarkers that are fundamental in the management of neovascular AMD [14,31].

In neovascular AMD, there are several biomarkers that are considered extremely important in the diagnosis and management of these patients:

- Intraretinal fluid (IRF) is characterized by the presence of round- or oval-shaped cysts within the inner retinal layers, appearing typically hyporeflective on OCT. IRF is more frequently associated with Type 2 and Type 3 MNV. Numerous studies suggest that IRF serves as a crucial negative prognostic biomarker, correlating not only with reduced visual acuity at baseline and less improvement after treatment, but also with a higher risk of fibrosis and atrophy development [32,33,34].
- Subretinal fluid (SRF) occurs when exudative fluid accumulates between the neuroretina and RPE. SRF is more frequently associated with Type 1 MNV. In contrast to IRF, SRF tends to indicate a more favorable prognosis. It is often associated with better visual acuity at baseline and after intravitreal therapy, as well as a reduced risk of atrophy [34,35]. However, it is important to note that the presence of SRF is considered as a negative biomarker in eyes with Type 3 MNV.
- Pigmented epithelium detachment (PED) occurs when there is a splitting between the RPE and Bruch’s membrane. The literature lacks consensus on the prognostic significance of PED. The latter aspect could be attributed to the existence of different types of PEDs, including fibrovascular, serous, drusenoid, and hemorrhagic, each potentially exerting distinct effects on visual acuity [34,35,36,37].
- Subretinal hyperreflective material (SHRM) refers to a hyperreflective material observed on structural OCT, situated beneath the neurosensory retina and above the RPE. SHRM could indicate various substances including fluid, blood, scar tissue, fibrin, vitelliform material, or neovascularization [38,39]. Previous studies have indicated that SHRM is a negative prognostic biomarker, correlating with a reduced response to anti-VEGF treatment and poorer visual outcomes [40,41,42].
- The disruption of the outer retinal layers refers to a notable OCT sign occurring when damage to the outer hyperreflective retinal layers is evident, including the ellipsoid zone (EZ) and external limiting membrane (ELM). Such disruptions in these hyperreflective bands have been associated with compromised visual acuity both at baseline and following anti-VEGF therapy [43,44,45].
- Retinal hyperreflective foci (HRF) are defined as hyperreflective spots on structural OCT, displaying a reflectivity akin to or higher than the retinal pigment epithelium (RPE), typically measuring between 20 to 40 microns and often exhibiting clear boundaries [46]. While intraretinal HRF may be the imaging surrogate of different cells/lesions, in AMD they are mostly associated with migrating RPE cells [47,48]. In a previous study, it was demonstrated that the number of HRF decreased after anti-VEGF therapy in responders, while they persisted in non-responders, and as a result their persistence despite treatment is considered a negative prognostic factor associated with poor VA [49]. Neuroretinal HRF were also suggested as an imaging indicator of inflammation in neovascular AMD, showing a decrease in number following effective anti-VEGF treatment [50]. Of note, HRF detection in neovascular AMD was considered a reliable predictor of poor visual prognosis after anti-VEGF treatment [51].

## 3. Artificial Intelligence in AMD

Artificial intelligence (AI) represents a new emerging tool in the medical field, offering expedited assistance in diagnosing, categorizing, and managing various prevalent diseases.

Image-based deep learning holds promise in swiftly analyzing vast image datasets, and this may be particularly beneficial in those medical fields characterized by a continual rise in patient numbers. Deep learning may aid in biomedical image interpretation and facilitate therapeutic decision-making across a number of medical specialties, including radiology, pathology, dermatology, and ophthalmology. Ophthalmology, especially the retinal field, heavily relies on image-based diagnostics such as OCT and color fundus photography. This reliance renders it highly suitable for the incorporation of computer-assisted diagnostic algorithms. Early applications of deep learning in retinal disorders utilized color fundus photography to classify conditions like diabetic retinopathy, AMD, and retinopathy of prematurity [52,53,54,55]. Subsequently, significant efforts have been dedicated to applying deep learning to OCT images. This focus stems from the recognition that the identification and quantification of the biomarkers of progression could greatly aid in monitoring patients undergoing treatment.

## 4. Deep Learning to Predict Progression from Intermediate to Neovascular AMD

In a previous important study, researchers developed a deep learning algorithm aimed at assessing the risk of disease progression from intermediate AMD to late AMD, including both geographic atrophy and neovascular AMD, in individuals with unilateral neovascular AMD. For the conversion to neovascular AMD, the authors evaluated different pathognomonic biomarkers and ranked them according to their prognostic significance. Among these biomarkers, the most relevant included drusen volume, area and average thickness, as well as the vertical extension of HRF in the outer nuclear layer (ONL). The study reported a 2-year area under the curve (AUC) for predicting neovascular AMD conversion of 0.68, with a specificity of 0.46 achieved at a sensitivity level of 0.80 [56].

Russakoff et al. [57], in a recent work, aimed to use deep learning algorithms to predict the conversion from early/intermediate AMD to neovascular AMD, based on OCT biomarkers. In particular, they analyzed OCT images of 71 eyes of patients with intermediate AMD and wAMD in the fellow eye, at baseline, in year 1 and year 2, using two different deep learning networks: for AMDnet the AUC was 0.89 on OCT b-scans and 0.91 on OCT volumes, while for VGG16 the AUC was 0.82 on b-scans and 0.87 at the volume level. Moreover, for non-progressors, areas around RPE seemed to have the larger impact on the final score, while for progressors choroid and under-RPE appeared to be more relevant [57].

Banerjee et al. [58] proposed a hybrid modeling approach, which incorporates the same platform radiomics, visual acuity and demographic data with deep learning: they first presented a sequential model for temporal prediction of exudation, from 3 to 21 months, in eyes with early or intermediate AMD, using the previous visits data. They obtained the best model performance at 3 months with an AUC of 0.96 ± 0.02, an AUC of 0.83 ± 0.04 at 6 months, and a decrease in the performance at 12 months (0.77 ± 0.06), followed by an improvement at 18 and 21 months (respectively AUC of 0.9 ± 0.06 and 0.97 ± 0.02) [58].

Similarly, Yim et al. [59]. proposed an AI system to predict conversion to exudative AMD in patients already treated in one eye for wAMD and with early–intermediate AMD in the fellow eye, during a 6 month follow up. This group showed that when using a single OCT scan for foreseeing conversion, the AI system outperformed five experts and matched one optometrist, plus, when the specialist had additional data, (OCT historic, fundus images, demographic and BCVA data) the model still showed a better performance than five experts and equal to one retinal specialist [59].

## 5. Deep Learning to Segment OCT Features in Patients with Neovascular AMD

Identifying and quantifying the OCT biomarkers linked to neovascular AMD may play a crucial role in determining patient outcomes in cases involving this condition [60,61].

In 2017, Lee and colleagues [62] were the first to utilize deep learning in OCT imaging, showing the capability of a deep learning model to differentiate between AMD and normal OCT images, yielding promising outcomes. Successively, Kermany and colleagues [63] utilized a deep learning algorithm to classify OCT images as normal or, alternatively, as affected by drusen, neovascular AMD, or diabetic macular edema. The latter may be considered as one of the first attempts to identify distinct OCT features in diseased eyes.

One of the first studies evaluating the different OCT biomarkers in neovascular AMD eyes was conducted by Schlegl and colleagues [64]. In the latter study, the authors introduced a fully automated deep learning approach not only for the detection but also for the quantification of fluid (i.e., IRF, SRF). Their model achieved an AUC of 0.93 for detecting IRF and an AUC of 0.98 for detecting SRF. The volumes of fluid assessed manually exhibited a strong correlation with the volumes segmented automatically, indicating excellent agreement between expert assessments and the AI model [64].

The number of OCT features detected and quantified in neovascular AMD eyes was successively increased in following studies. Lee et al. [65]. constructed a dataset including 930 B-scans from 93 eyes of 93 patients with neovascular AMD and validated a 2D trained network, showing a high agreement with manual labeling in the detection of IRF, SRF, SHRM and DEP in 930 OCT B-scans.

Subsequently, a three-dimensional CNN network algorithm known as 3D U-NET was introduced for the segmentation of retinal OCT images. This model took into account spatial–temporal correlations between adjacent scans. The 3D U-NET demonstrated superior segmentation accuracy, achieving a remarkable overall accuracy of 99.56%. Particularly noteworthy were its results in retinal fluid segmentation, with a Kappa coefficient of 98.47% and an F1 score of 95.50%. These outcomes closely resembled the manually labeled annotations provided by ophthalmology specialists [66].

The amount of OCT biomarkers detected and quantified using a deep learning 3D segmentation network was expanded in a study by Moraes et al. [67]. They assessed 2966 OCT scans from the Moorfield AMD database, comprising eyes with treatment-naive neovascular AMD yet to undergo anti-VEGF therapy or previously treated eyes. The latter study analyzed various segmented features, including central foveal thickness, RPE atrophy, IRF, SRF, SHRM, HRF, drusenoid, fibrovascular and serous PED. Except for drusen, the eyes first treated generally exhibited greater volumes across all analyzed features. Fibrovascular PED and SHRM volumes showed a positive correlation with each other and with SRF volumes, but a weaker correlation with IRF volume. HRF demonstrated a strong volumetric correlation with IRF. Regarding VA, all biomarkers exhibited a negative volumetric correlation with VA in treatment-naive eyes, while only SHRM, RPE atrophy, central foveal thickness, and IRF showed a statistically significant negative correlation in second treated eyes. Notably, SHRM volume displayed the most substantial negative correlation with VA for both first and second treated eyes, and IRF showed a stronger association with VA compared to SRF, highlighting the importance of distinguishing between different fluid types [67].

Liefers et al. [68], in a recent study, proposed a deep learning segmentation model for 13 biomarkers commonly found in neovascular AMD. They compared the performance of the model with the performance obtained from four experienced graders. Overall, the model performed better than the manual grading in the quantification of IRF, SHRM, RPE loss and ellipsoid zone loss, while the manual labeling was slightly better in the quantification of drusen and drusenoid PED. For the other features, the manual and deep learning-based assessments performed similarly. This study represents a pioneering effort in the comprehensive analysis of multiple OCT features, extending beyond fluid-based biomarkers to include features of atrophy such as RPE loss and EZ loss. Remarkably, this study illustrates that the model’s performance is comparable to, and occasionally surpasses, that of experienced human graders.

Our group has extensively worked on the application of deep learning to quantify critical OCT biomarkers associated with neovascular AMD. First, we performed a pilot study on 50 eyes (50 patients) to develop and validate a deep learning algorithm for automated IRF, SRF and neovascular PED segmentation in neovascular AMD [69]. In the latter study, we validated a deep learning algorithm for automated IRF and SRF segmentation in neovascular AMD. Successively, we adopted a methodology similar to that used in the aforementioned paper, although the current investigation was conducted on a large real-world dataset and an extended feature set [70]. Three-hundred OCT volumes from subject eyes with neovascular AMD were collected. The images were manually segmented for the presence of five crucial OCT features: IRF, SRF, SHRM, drusen/drusenoid PED, and neovascular PED. A deep learning architecture based on a U-Net was trained to perform automatic segmentation of these retinal biomarkers and evaluated on the sequestered data. The model obtained a mean (±SD) AUC of 0.93 (±0.04) per slice and 0.88 (±0.07) per volume for fluid detection. The correlation score (R^2^) between automatic and manual segmentation obtained by the model resulted in a mean (±SD) of 0.89 (±0.05). The mean (±SD) 2-D correlation score was 0.69 (±0.04). The mean (±SD) Dice score resulted in 0.61 (±0.10). Overall, this model, for five features related to neovascular AMD, performs at the level of experienced graders.

## 6. Deep Learning to Predict Anti-VEGF Treatment in Patients with Neovascular AMD

Bogunovic et al. [71] introduced a machine learning approach aimed at predicting low and high injection requirements in patients with neovascular AMD undergoing a 2-year pro re nata (PRN) treatment regimen. The objective was to evaluate biomarker presence during the initial visits following the initiation of anti-VEGF therapy, including baseline, 1-month follow-up, and 2-month follow-up visits. They employed a convolutional neural network (CNN) trained on 20,000 OCT B-scans. Their findings revealed that the AUCs for predicting the categories steadily increased over time, starting at 0.60 at baseline, reaching 0.68 at month 1, 0.70 at month 2 for low treatment requirements, and 0.61 at baseline, escalating to 0.74 at month 1, and 0.77 at month 2 for high treatment requirements. Thus, the retinal morphology at baseline before intravitreal injection appears to be less predictive of future treatment needs compared to the retinal morphology after the initial anti-VEGF treatment. According to this study, the three most crucial features in predicting the number of injections required are: SRF in the central 3 mm at month 2, inner retinal thickness in the fovea at month 1, and inner retinal thickness in the central 3 mm at month 2. The model’s predictive performance was comparable to that of human graders for low requirements, and 50% better than expert grading for the high requirements category.

Similarly, Pfau et al. [72] utilized three distinct machine learning algorithms to anticipate the frequency of intravitreal treatments over the subsequent 12 months for neovascular AMD patients undergoing PRN or treat-and-extend treatment regimens. The mean absolute error (MAE) predictions were 2.76 injections per year (range: 2.39–3.14) using lasso regression, 2.74 injections per year (range: 2.38–3.11) using principal component regression, and 2.60 injections per year (range: 2.25–2.96) for random forest regression. All models predicted a higher number of injections than necessary for the lower treatment group and underestimated the required injection frequency for the higher treatment group.

Feng et al. [73] introduced a deep learning algorithm designed to assess the effectiveness of anti-VEGF treatment in patients with neovascular AMD, solely relying on OCT images before treatment initiation. They employed the ResNet-50 architecture, pretrained on ImageNet, and trained it using four distinct datasets. A comparative analysis against other established models revealed that their model achieved the highest AUC (0.81), indicating its superior capability in predicting the effectiveness of anti-VEGF treatment. Additionally, they observed improved outcomes when utilizing OCT full images compared to solely focusing on pathological regions [73].

Two recent studies [74,75] have introduced a model for predicting OCT post-therapeutic images by employing generative adversarial network (GAN) technology. Initially, the model was trained using both pre- and post-therapeutic images, after which it generated synthetic OCT scans for comparison with actual scans. Both of these studies demonstrate the significant potential of GAN in generating post-anti-VEGF OCT scans with high accuracy and quality. Similarly, Moon et al. [76] developed an AI model aimed at predicting anatomical treatment outcomes specific to anti-VEGF agents in neovascular AMD, assisting clinicians in selecting the most appropriate agent for individual patients. This retrospective study involved patients diagnosed with neovascular AMD who underwent three loading injections of either ranibizumab or aflibercept, training the utilized OCT images with an attention GAN model. To assess the AI model’s performance, the sensitivity and specificity in predicting the presence of retinal fluid post-treatment were calculated for the AI model, an experienced examiner (Examiner 1), and a less experienced examiner (Examiner 2). The training set comprised 1684 OCT images from 842 patients (419 ranibizumab-treated and 423 aflibercept-treated), while testing employed images from 98 patients. For patients treated with ranibizumab, the AI model demonstrated a sensitivity and specificity of 0.615 and 0.667, respectively, while Examiner 1 showed 0.385 sensitivity and 0.861 specificity, and Examiner 2 exhibited 0.231 sensitivity and 0.806 specificity. In aflibercept-treated patients, the AI model achieved a sensitivity and specificity of 0.857 and 0.881, respectively, compared to 0.429 sensitivity and 0.976 specificity for Examiner 1, and 0.429 sensitivity and 0.857 specificity for Examiner 2. Furthermore, in 18.5% of cases, the fluid status differed between synthetic post-treatment images of ranibizumab and aflibercept. The AI model utilizing GAN showed potential in predicting agent-specific short-term treatment outcomes, with higher sensitivity than human examiners. Additionally, variations in efficacy were observed in fluid resolution between the anti-VEGF agents, underscoring the potential of AI in personalized medicine for neovascular AMD patients [76].

Finally, Ursula Schmidt-Erfurth et al. [77] used machine learning regression using random forests to predict final BCVA at 12 months in patients with active neovascular AMD undergoing anti VEGF treatment with ranibizumab. In the latter study, the authors revealed that BCVA at 3 months and, among imaging biomarkers, the horizontal extension of IRF both centrally within 3 mm and in the parafoveal area were the most significant predictors for the final BCVA outcome.

## 7. Conclusions

This review highlights the emerging applications of deep learning in neovascular AMD.

Considering the continual aging of the population, the prevalence of neovascular AMD patients is steadily increasing, with the latter necessitating a corresponding rise in ophthalmology examinations. As multimodal imaging techniques evolve, deep learning may play a crucial role in extracting relevant data, processing information, and alleviating the workload of physicians, thereby enhancing patient management and therapeutic decision-making.

The integration of deep learning and OCT image analysis holds potential for predicting the conversion to neovascular AMD and promptly referring patients to specialists for the expedited initiation of anti-VEGF therapy, thus mitigating permanent damage and optimizing visual outcomes and quality of life. Additionally, the analysis of imaging biomarkers using deep learning models promises to furnish more precise functional prognoses, enabling clinicians to tailor patient visit schedules optimally, thereby maximizing resource utilization and improving treatment outcomes.

## Data Availability

Not applicable.
